# 27. Immunologic Hyporesponsiveness with Subsequent Dosing of Meningococcal Vaccines: Re-Evaluating the Current Paradigm

**DOI:** 10.1093/ofid/ofab466.229

**Published:** 2021-12-04

**Authors:** Jamie Findlow, Paul Balmer

**Affiliations:** 1 Pfizer Ltd, Tadworth, England, United Kingdom; 2 Pfizer Inc, Collegeville, Pennsylvania

## Abstract

**Background:**

Immunologic hyporesponsiveness (HyR) is considered as an inability to mount immune responses to vaccination of at least the same degree as earlier doses. For meningococcal vaccines, HyR has classically been associated with unconjugated but not conjugated polysaccharide (PS) vaccine dosing, but the clinical relevance is unclear.

**Methods:**

To characterize meningococcal vaccine HyR, a PubMed search was conducted without date limits as follows: (hyporespons*) AND (meningococcal) AND (vaccine OR mechanism OR MOA OR causes). Papers from the authors’ files, including HyR insights with other vaccines, were included.

**Results:**

Classic HyR with repeat unconjugated PS vaccine (MPV) dosing is thought to be associated with memory B-cell (BC) depletion, causing reduced responses on redosing with the same PS. This lack of immunologic memory and interference is seen years after MPV dosing across age groups. As data is added, other examples seem to fit the HyR definition but differ from the classical mechanism and its implications. First, passively transferred maternal antibodies (Abs) may interfere with neonatal adaptive immune response and ultimately those of childhood vaccination by binding to vaccine antigen (Ag) and inhibiting Ab production. Second, multiple dose schedules of meningococcal conjugate vaccines can show reduced responses to later doses in the series but memory is still established and amnestic booster response later achieved. Finally, carrier-induced epitopic suppression, occurring when PS Ag epitopes presented on a protein carrier are inhibited by prior/concurrent dosing with the same carrier, has also been reported. These 3 examples of alternative HyR mechanisms are not associated with memory BC depletion but are likely due to high circulatory Ab levels reducing responses, which is transient, reduces with Ab waning, immunologic memory remains intact, and is not clinically significant.

**Conclusion:**

This literature review identified HyR mechanisms other than the classic mechanism associated with memory BC depletion that may account for decreased immune response to subsequent vaccination. Understanding the type of HyR observed with meningococcal vaccines is crucial, as these mechanisms vary in terms of potential clinical significance and the duration of their impact.

**Disclosures:**

**Jamie Findlow, PhD**, **Pfizer** (Employee, Shareholder) **Paul Balmer, PhD**, **Pfizer** (Employee, Shareholder)

